# Establishment of Chemotherapy Prediction Model Based on Hypoxia-Related Genes for Oral Cancer

**DOI:** 10.7150/jca.96654

**Published:** 2024-08-13

**Authors:** Chuhuan Zhou, Hanqi Jia, Nan Jiang, Jingli Zhao, Xinrong Nan

**Affiliations:** 1 Shanxi Medical University School and Hospital of Stomatology, Taiyuan, 030001, China.; 2 Shanxi Province Key Laboratory of Oral Diseases Prevention and New Materials, Taiyuan, 030001, China.; 3 The First Affiliated Hospital of Shanxi Medical University, Taiyuan, 030001, China.

**Keywords:** Oral cancer, Machine learning, ALDOA, Hypoxia

## Abstract

**Purpose:** Identify the hypoxia genes related to chemotherapy resistance of oral cancer, and construct a chemotherapy response model by machine learning algorithm.

**Methods:** 72 oral cancer patients with complete chemotherapy records and chemotherapy reactions were screened from the Cancer Genome Atlas (TCGA) database. According to the chemotherapy reactions, they were divided into chemotherapy sensitive group and chemotherapy resistant group. The differential genes were screened by Limma package. Then the chemotherapy response gene were screened by univariate analysis. Based on the gene expression profile of chemotherapy response, four machine learning algorithms were used to construct the prediction model of chemotherapy response. The core genes were screened by lasso regression analysis. Finally, the prognosis and immune infiltration of the core genes were analyzed. The results were verified by immunohistochemistry (IHC).

**Results:** We obtained 22 hypoxia related differential genes. Univariate analysis found 6 Chemotherapy response genes. Machine learning algorithms show that XGBoost have the best predictive performance for chemotherapy response. ALDOA is the core gene of chemotherapy resistance.

**Conclusions:** Successfully constructed a chemotherapy prediction model for oral cancer by machine learning algorithm. Under hypoxia, the high expression of ALDOA is associated with chemotherapy resistance in oral cancer.

## Introduction

Oral squamous cell carcinoma (OSCC) is one of the common malignant tumors in the head and neck. Although many studies have been devoted to improving the survival rate of OSCC patients, the five-year survival rate is still 50% -60% 1, 2. Chemotherapy refers to the use of chemotherapeutic drugs to kill cancer cells to achieve the purpose of treatment. It is one of the important components of systemic treatment for OSCC patients. However, the emergence of chemotherapy resistance is one of the important causes of death in cancer patients 3. The generation of chemotherapy resistance is mediated by many factors 4-9. A large number of studies have shown that there is a close relationship between hypoxia and chemotherapy resistance. Hypoxia is one of the typical signs of solid tumors 10. Hypoxia can affect the overall survival of patients by regulating the tumor microenvironment. At present, studies have shown that hypoxia can lead to the tolerance of cancer patients to chemotherapy drugs through a variety of signaling pathways such as apoptosis, autophagy, DNA damage, mitochondrial activity, p53 and drug efflux. Therefore, the targeting of hypoxia related genes may improve the chemotherapy resistance caused by hypoxia 11.

With the rapid development of genomics, the huge data content makes the traditional analysis methods unable to meet the needs of researchers' data processing. Bioinformatics analysis methods based on computer data processing are widely used in various fields of research. Bioinformatics in the broad sense refers to the use of mathematical, statistical and computational methods to process and analyze biological data 12. In this study, we identified the differentially expressed genes related to hypoxia chemotherapy by bioinformatics, and then conducted lasso regression analysis on the selected genes to further establish ALDOA as the key gene of chemotherapy resistance. Aldolase is a key enzyme in glycolysis, which can be divided into three types: ALDOA, ALDOB, and ALDOC 13. ALDOA plays a crucial role in regulating cell shape and activity, skeletal muscle contraction, organization of actin filaments, and ATP biosynthesis. Research has indicated that ALDOA exhibits elevated expression levels in lung adenocarcinoma, hepatocellular carcinoma, and head and neck squamous cell carcinoma compared to normal tissue. An increase in ALDOA expression is often correlated with a poor prognosis for patients 14. While previous studies have investigated the relationship between ALDOA and oral cancer 15, 16, primarily focusing on prognostic implications. This study builds upon existing prognostic analyses and delves further into elucidating the connection between ALDOA and chemotherapy responsiveness. We identify hypoxia gene ALDOA as a core gene of chemoresistance in oral cancer. By targeting ALDOA, it is expected to improve the sensitivity of tumor tissue to chemotherapy under hypoxia. Meanwhile, this study also uses machine learning algorithm to build chemotherapy response prediction model. Machine learning can build a prediction model by analyzing data, and can continuously improve itself by organizing the existing knowledge structure 17. The chemotherapy response prediction model we constructed can make a preliminary prediction of whether patients are resistant to chemotherapy, so as to avoid the excessive use of chemotherapy. Finally, the results of the bioinformatics analysis were validated using immunohistochemistry techniques.

## Materials and Methods

### Participants

The clinical data and transcriptome data of patients were downloaded from TCGA database, and 72 oral cancer patients with complete chemotherapy records and chemotherapy reactions were selected according to the tumor anatomical sites. With the approval of the hospital ethics committee, we obtained tissue wax blocks from 21 patients with oral cancer from the Department of pathology of the first hospital of Shanxi Medical University, the time span is December 2021 and December 2023. All patients received chemotherapy. The inclusion criteria were as follows: 1) the tumor lesions were primarily located in the mouth and were diagnosed as squamous cell carcinoma by pathological examination; 2) No other treatment before admission; 3) All patients received systematic and standardized treatment according to the latest NCCN guidelines for the diagnosis and treatment of oral cancer; 4) All patients had complete pathological data and follow-up data; 5) No other serious history of systemic diseases or malignant tumors; Exclusion criteria: 1) the primary tumor was located in the mouth, and was diagnosed as salivary gland cancer, sarcoma, jaw cancer, lymphoma, malignant melanoma and other non squamous cell carcinoma types by pathological examination; 2) Before admission, he had received surgical treatment, radiotherapy or drug chemotherapy in other hospitals; 3) Patients who give up treatment or do not receive standardized treatment for other reasons.

### Data download and processing

ID conversion was performed on the downloaded transcriptome data, and the data were standardized. A total of 546 samples were included, including 502 tumor tissues and 44 normal tissues. Meanwhile, the clinical data were processed, and 72 cases with complete chemotherapy records and chemotherapy reactions were selected. We defined the patients with complete remission and partial remission as the chemotherapy sensitive group, and the patients with stable disease and disease progression as the chemotherapy resistant group, including 42 patients in the chemotherapy sensitive group and 30 patients in the chemotherapy resistant group.

### Identification of hypoxia related differential genes

The significant difference genes between chemotherapy sensitive group and chemotherapy resistant group were analyzed by R software limma package. The screening condition of difference genes was |LogFc | ≥ 1,* P* adjust<0.05. Meanwhile, the gene set related to hypoxia was found from the GSEA website (https://www.gsea-msigdb.org/gsea/index.jsp). Then we intersected the significant difference genes with 200 hypoxia related genes to obtain the hypoxia related difference genes.

### Gene enrichment analysis

We divided the samples into chemotherapy sensitive group and chemotherapy tolerant group. Meanwhile, we set the genome scope according to the transcriptome expression profile and phenotype grouping, and conducted 1000 resampling to obtain the phenotypes enriched by the two groups of gene sets.

### Univariate analysis

Univariate analysis of genes related to hypoxia was carried out, and six chemotherapy response gene were obtained (*P*<0.05). Meanwhile, the hazard ratio value and 95% confidence interval of patients were calculated, and the related results were displayed in the form of forest map.

### K-M (Kaplan-Meier) survival curve

Sangerbox (http://www.sangerbox.com/tool) is a platform for online data analysis based on the network 18, we use this platform for visual analysis, which can visualize various data. We calculate the risk scores of the six genes screened by univariate analysis, calculate the best cut-off value of the risk score using maxstat R package, and divide the patients into high-risk group and low-risk group according to the best cut-off value. Meanwhile, the survfit function was also used to analyze the prognosis difference between the two groups, and the logrank test was used to evaluate the significance of the prognosis difference between the two groups.

### Construction of a chemotherapy response prediction model

We randomly divided patients into a modeling group and a validation group in a 7:3 ratio. Simultaneously incorporating patient chemotherapy response and expression profile data of six genes (ALDOA, VEGFA, IER3, LOX, KLHL24, and TGFB3), four machine algorithms including decision tree, random forest, support vector machine, and XGBoost were applied to construct the chemotherapy response model. Meanwhile, AUC values were calculated to evaluate the accuracy of the four models.

### Core gene identification

In order to further determine the core genes related to chemotherapy resistance, glmnet R package was used to include three variables including survival time, survival status and gene expression data. The six genes obtained from univariate analysis were analyzed by lasso Cox regression analysis. Meanwhile, a 5-fold cross validation was set to obtain the best model. Finally, we got two genes (the screening condition was that lambda value was equal to 0.18), The HPA (Human Protein Atlas) is a comprehensive database grounded in proteomics, transcriptomics, and systems biology, encompassing gene expression atlases for a multitude of tissues, cells, and organs 19. The HPA database collects the expression of genes in various cancer cell lines, through HPA database, we can know the expression of core genes in cell lines. Meanwhile, we compared the gene expression levels of the two groups in the sensitive and resistant groups, and selected the genes with the most obvious difference. Based on the above information and lasso regression analysis results, we finally determined the core genes.

### Immune infiltration and analysis of clinical prognosis

Based on the median value of mRNA expression of ALDOA, patients were divided into low and high expression groups. Cibersort method was used to determine the infiltration abundance of 22 kinds of immune cells in the two groups. Meanwhile, five variables including age, gender, T stage, N stage and Pathologic stage were included. Age was divided into “≤ 60 years old” and “>60 years old”, gender was divided into “female” and “male”, T stage was divided into “T1+T2” and “T3+T4”, N stage was divided into “N0+N1” and “N2+N3”, The stage was divided into “stage II+III” and “stage IV”. We investigated the relationship between ALDOA expression and clinical prognosis.

### Efficacy evaluation

This study is a retrospective study. All patients underwent magnetic resonance imaging before and after chemotherapy. We obtained the magnetic resonance images of 21 patients with oral cancer from the imaging department of the hospital. According to the World Health Organization and RECIST standard 20, the objective response to treatment was divided into four categories: complete response (CR), partial response (PR), stable disease (SD) and progress disease (PD). CR showed that all the target lesions disappeared, and the short diameter of pathological lymph nodes was<10 mm; PR is the reduction of the total length and diameter of the target lesion ≥ 30%; SD means that the total length and diameter of the target lesion decreased but did not reach PR or increased but did not reach PD; PD is defined as an increase of ≥ 20% in the total length and diameter of the target lesion, and its absolute value increases by more than 5 mm. The occurrence of new lesions is also regarded as PD. Two MRI professionals with more than 5 years' working experience outlined the scope of the target lesion and resolved the differences by consensus. Patients with CR+PR were divided into chemotherapy sensitive group, and patients with SD+PD were divided into chemotherapy resistant group. Based on this standard, there were 15 patients in the chemotherapy sensitive group and 6 patients in the chemotherapy resistant group.

### Immunohistochemistry

Tissue wax blocks from 21 chemotherapy patients were provided by the pathology department of the hospital. The wax block originates from tumor tissue surgically resected after chemotherapy. Tissue sections were dewaxed in xylene for 10 minutes and then hydrated for 5 minutes in ethanol with decreasing concentrations (100%, 95%, 85% and 75% ethanol). Antigen repair with sodium citrate buffer for 2 minutes. Sections were soaked in incubated with 3% hydrogen peroxide for 10 minutes. Following this, drop primary antibody, they were incubated overnight at 4 °C in a refrigerator. After removal of the primary antibody, the secondary antibody was added and incubate at 37 °C temperature for 1H. Upon removal of the secondary antibody, and finally conduct DAB color development and hematoxylin staining.

ALDOA was mainly expressed in the cytoplasm. ALDOA antibody for IHC staining was obtained from proteintech (67453-1-Ig). The IHC scores were independently assessed by two experienced pathologists. The results were determined based on the positive cell rate score and staining intensity score. A: Staining intensity (0 = negative staining, 1 = weak staining, 2 = moderate staining, and 3 = strong staining. B: Positive cell proportion (1: ≤ 25%, 2: 26-50%, 3: 51-75%, and 4: > 75%) The IHC score was calculated as positive cells score × staining intensity score. IHC score ≥ 4 is positive.

### Statistical analysis

The counting data were analyzed by Fisher exact probability test, and described by rate and percentage. The measurement data were first tested for normal distribution by Shapiro Wilk statistics. If it was normal distribution, *t* test was used when the variance was homogeneous, and *F* test was used when the variance was uneven; If it is non normal distribution, Wilcoxon rank sum test is used. Rank sum test was used for rank data. Pearson correlation coefficient was used for correlation analysis. All statistical results are expressed inα=0.05 was the test level, *P*<0.05 was the difference was statistically significant.

## Results

### Clinical characteristics of patients

**Figure 1:**
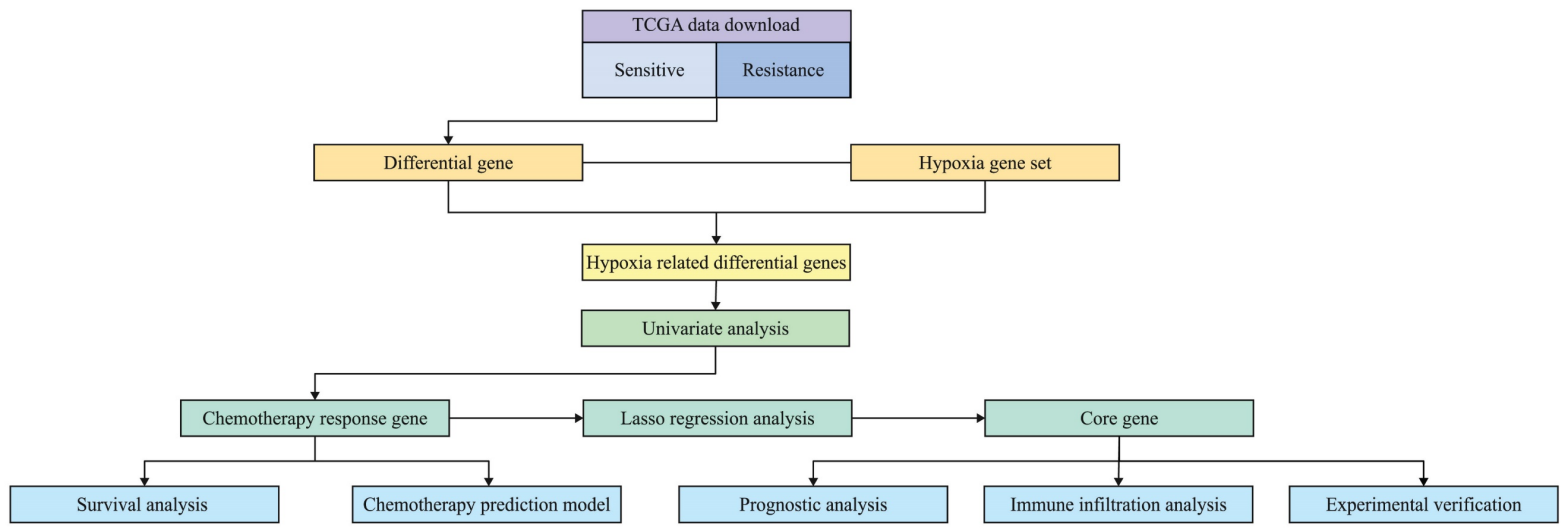
Flow chart of the experiment.

Figure 1 is the flow chart of this study. The clinical data and transcriptome data of patients were downloaded from TCGA database, and the patients with complete chemotherapy records and chemotherapy reactions were screened. A total of 72 patients were included. The clinical characteristics of 72 patients with oral cancer are shown in Table 1. According to the median expression of ALDOA, the patients were divided into low expression group and high expression group. The average age of high expression group was 58.80 years old. Most patients were concentrated in “T3+T4” and “N2+N3” subgroups, which suggested that high expression of ALDOA was associated with poor prognosis.

### Hypoxia related differential genes

**Figure 2:**
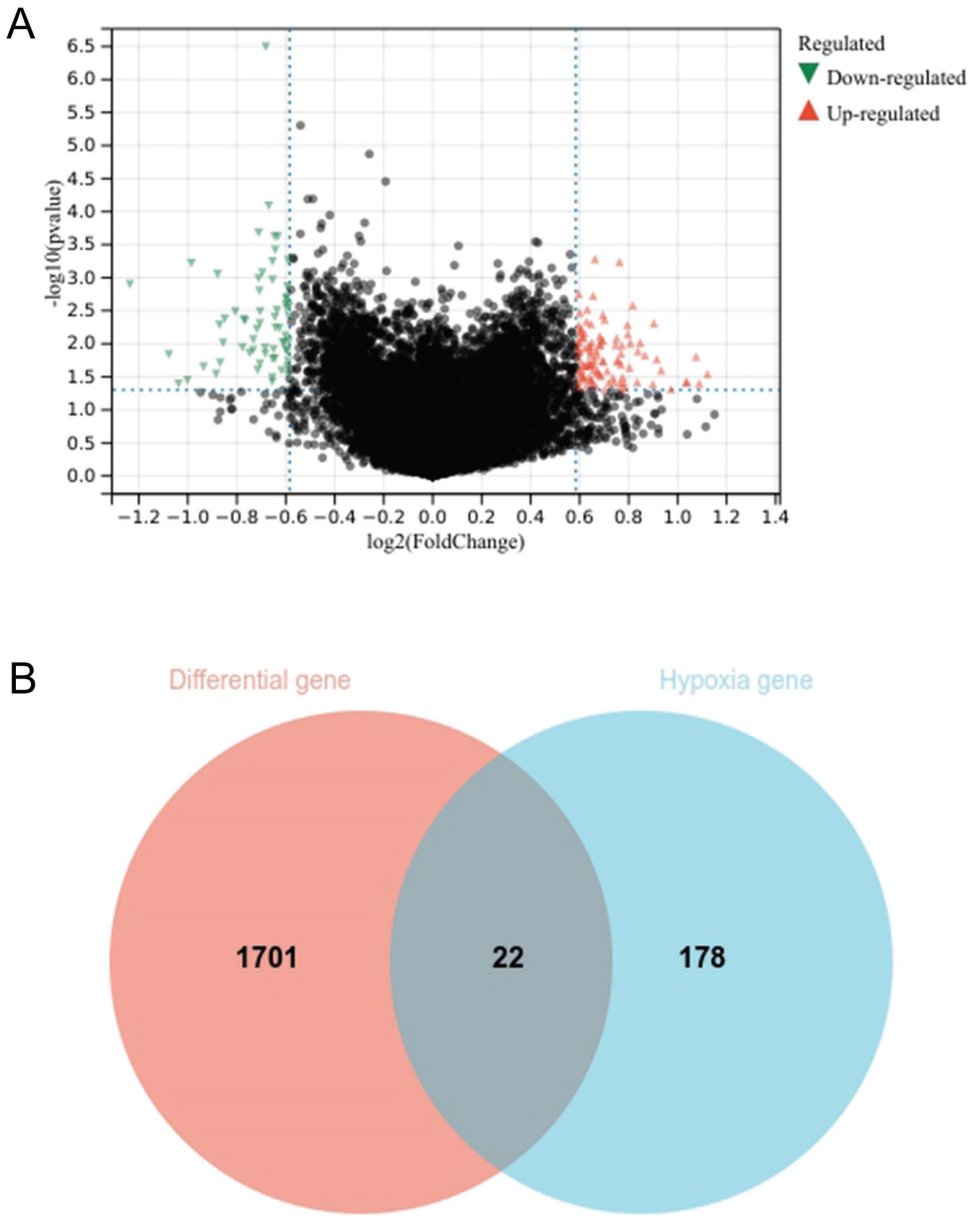
Volcano plot of differential genes between chemotherapy sensitive group and chemotherapy resistant group. Red represents up-regulated genes and green represents down-regulated genes (A); Intersection genes of differential genes and hypoxia gene set (B).

The genes of chemotherapy sensitive patients and chemotherapy resistant patients were compared by limma package. A total of 1723 differential genes were screened, including 856 up-regulated genes and 867 down regulated genes (Fig. 2A). The setting conditions were 1.2-fold difference, *P*<0.05. The differential genes were crossed with 200 hypoxia genes to obtain 22 genes (Fig. 2B).

### Gene enrichment analysis and chemotherapy response gene

**Figure 3:**
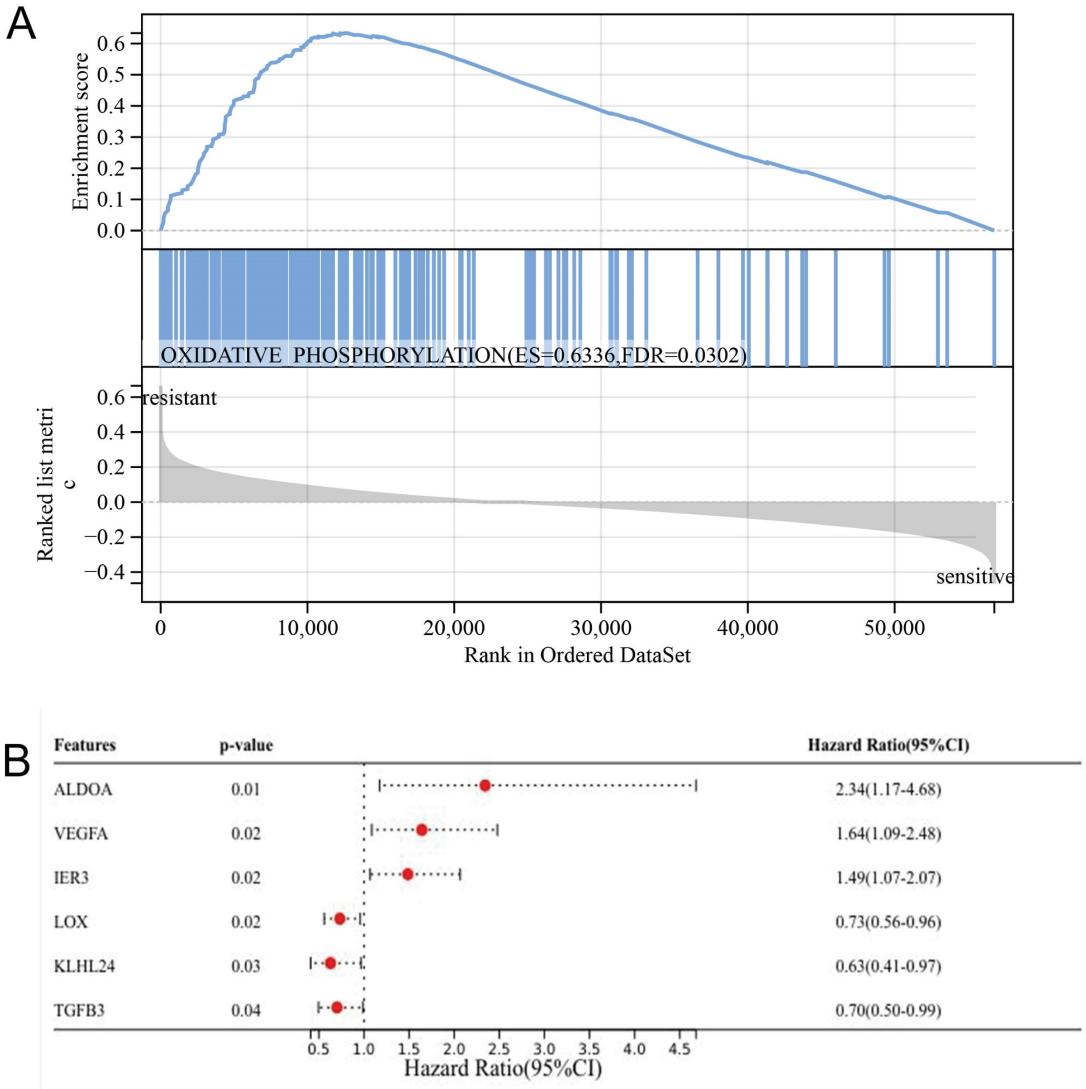
Graph of gene enrichment analysis results (A); Univariate analysis results (B).

Gene enrichment analysis has revealed that oxidative phosphorylation is predominantly associated with chemotherapy resistance, with ES value of 0.6336 and FDR value of 0.0302 (Fig. 3A). Univariate analysis has pinpointed six genes ALDOA, VEGFA, IER3, LOX, KLHL24, and TGFB3 as key determinants of chemotherapy response (Fig. 3B). Through the prognosis heat map, we can know that the high expression of ALDOA, VEGFA and IER3 has a low survival rate, while the high expression of LOX, KLHL24 and TGFB3 has a high survival rate, indicating that the three genes of ALDOA, VEGFA and IER3 are bad prognosis genes, while the three genes of LOX, KLHL24 and TGFB3 are protective factors.

### K-M survival curve

**Figure 4:**
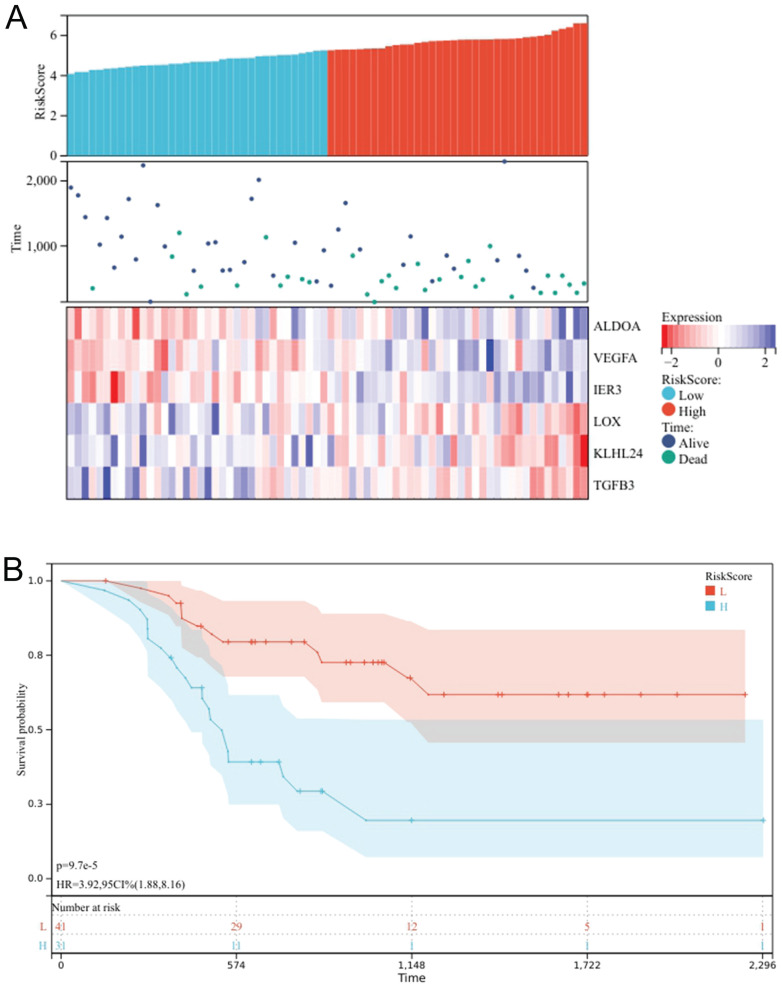
Prognostic Heatmap of chemotherapy response genes (A); Survival analysis chart of high and low risk groups (B).

A prognostic heat map indicates that high expression levels of ALDOA, VEGFA, and IER3 are correlated with a lower survival rate, suggesting that these three genes are indicative of poor prognosis. Conversely, high expression levels of LOX, KLHL24, and TGFB3 are associated with a higher survival rate, indicating that these three genes act as protective factors (Fig. 4A), The risk scores of six genes were calculated, and the patients were divided into high-risk group and low-risk group according to the best cut-off value. Through the K-M survival curve, we can know that the high-risk group has a lower survival rate, and the low-risk group has a better prognosis, *P*<0.05, the difference was statistically significant (Fig. 4B). These results indicate that these six genes can be used as prognosis prediction genes for patients with oral cancer undergoing chemotherapy.

### Results of constructing a chemotherapy response prediction model

**Figure 5:**
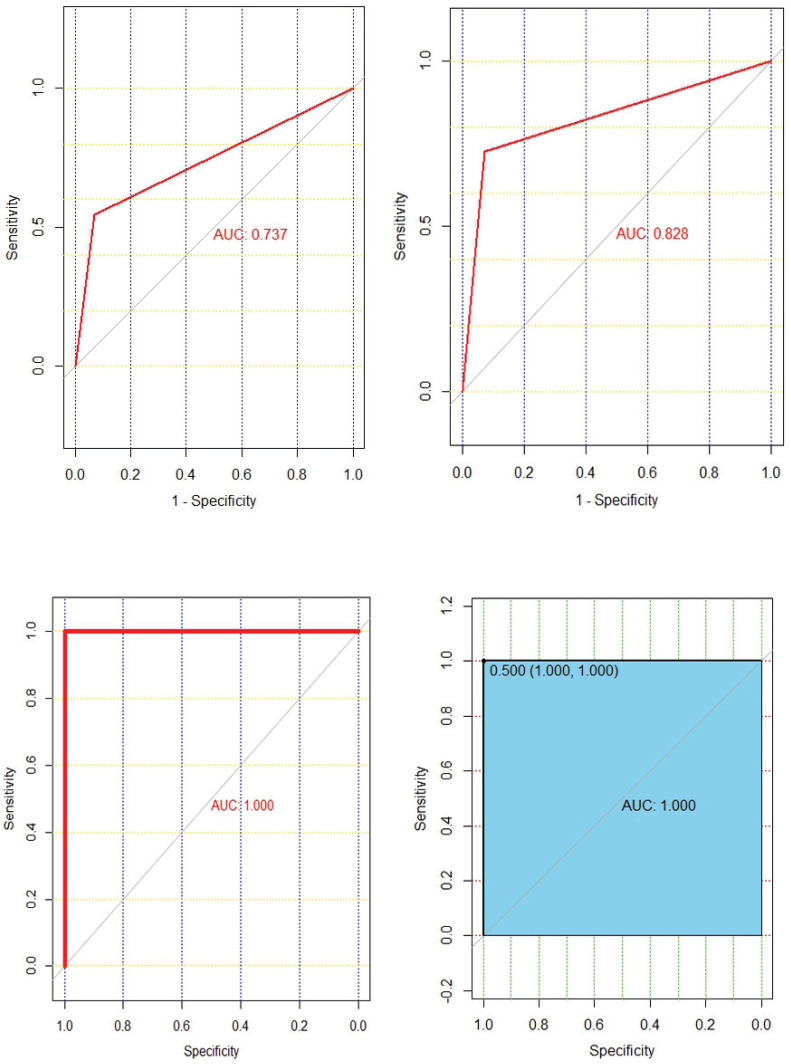
The ROC curves of decision trees, support vector machines, random forests, and XGBoost models are shown in the top left, top right, bottom left, and bottom right, respectively.

Chemotherapy response prediction model was successfully constructed using four machine learning algorithms. The AUC values of decision tree, support vector machine, random forest, and XGBoost models are 0.737, 0.828, 1.000, and 1.000, respectively (Fig. 5). The AUC values of the validation group were 0.527, 0.625, 0.676, and 0.741, respectively (Supplementary Figure 1). In summary, we can know that XGBoost algorithm has the highest accuracy in predicting chemotherapy response.

### Determination of core gene ALDOA

**Figure 6:**
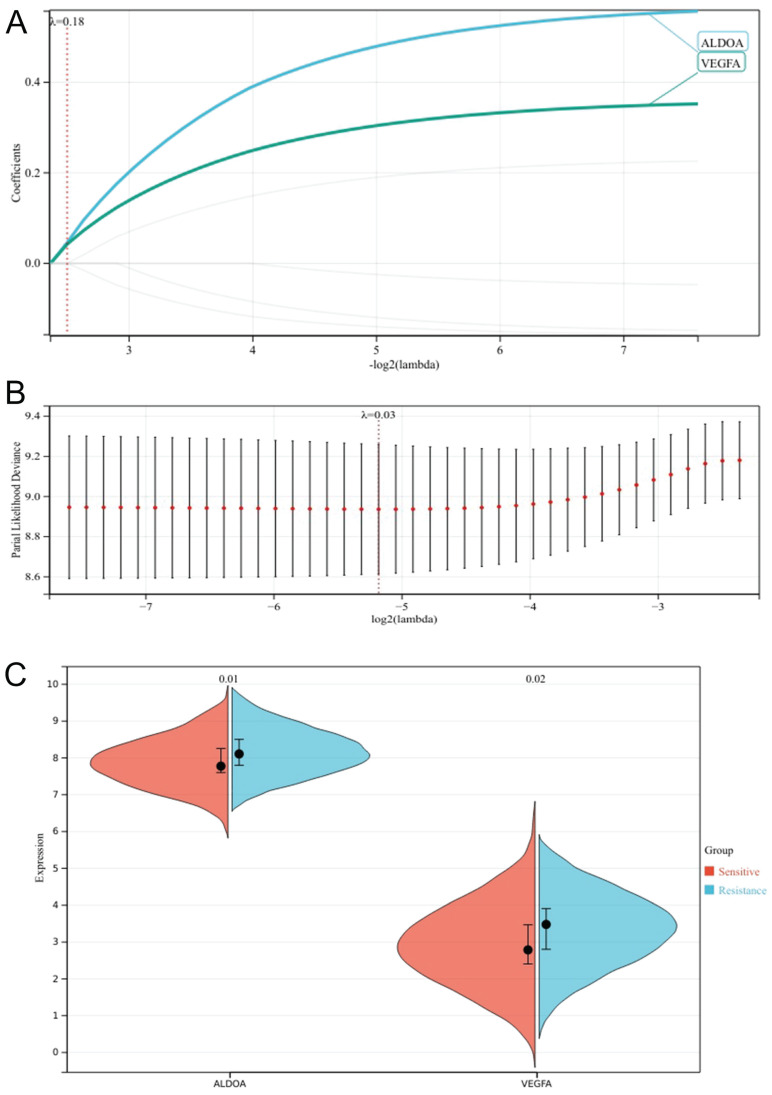
Lasso regression analysis cross validation curve (A); Lasso coefficient path chart (B); Faceted violin plot of VEGFA versus ALDOA expression (C).

We set the lammb value to 0.18, and finally got two genes that were most related to the poor prognosis of patients, VEGFA and ALDOA (Fig. 6A,B). Meanwhile, we compared the expression differences of the two genes in the chemotherapy sensitive group and the chemotherapy resistant group, and found that the difference in the expression of ALDOA (*P*=0.01) was greater than that of VEGFA (*P*=0.02) (Fig. 6C). In the HPA database, we searched the expression of the two genes in the head and neck tumor cell lines, and found that the cell lines with high expression of ALDOA were PE/CA-PJ49, the cell lines with high expression of VEGFA were SNU-1214 (Supplementary Figure 2), ALDOA were highly expressed in OSCC lines, and VEGFA were highly expressed in laryngeal squamous cell lines. Therefore, we determined that ALDOA was the core hypoxia gene of chemotherapy resistance in oral cancer.

### Immune infiltration analysis

**Figure 7:**
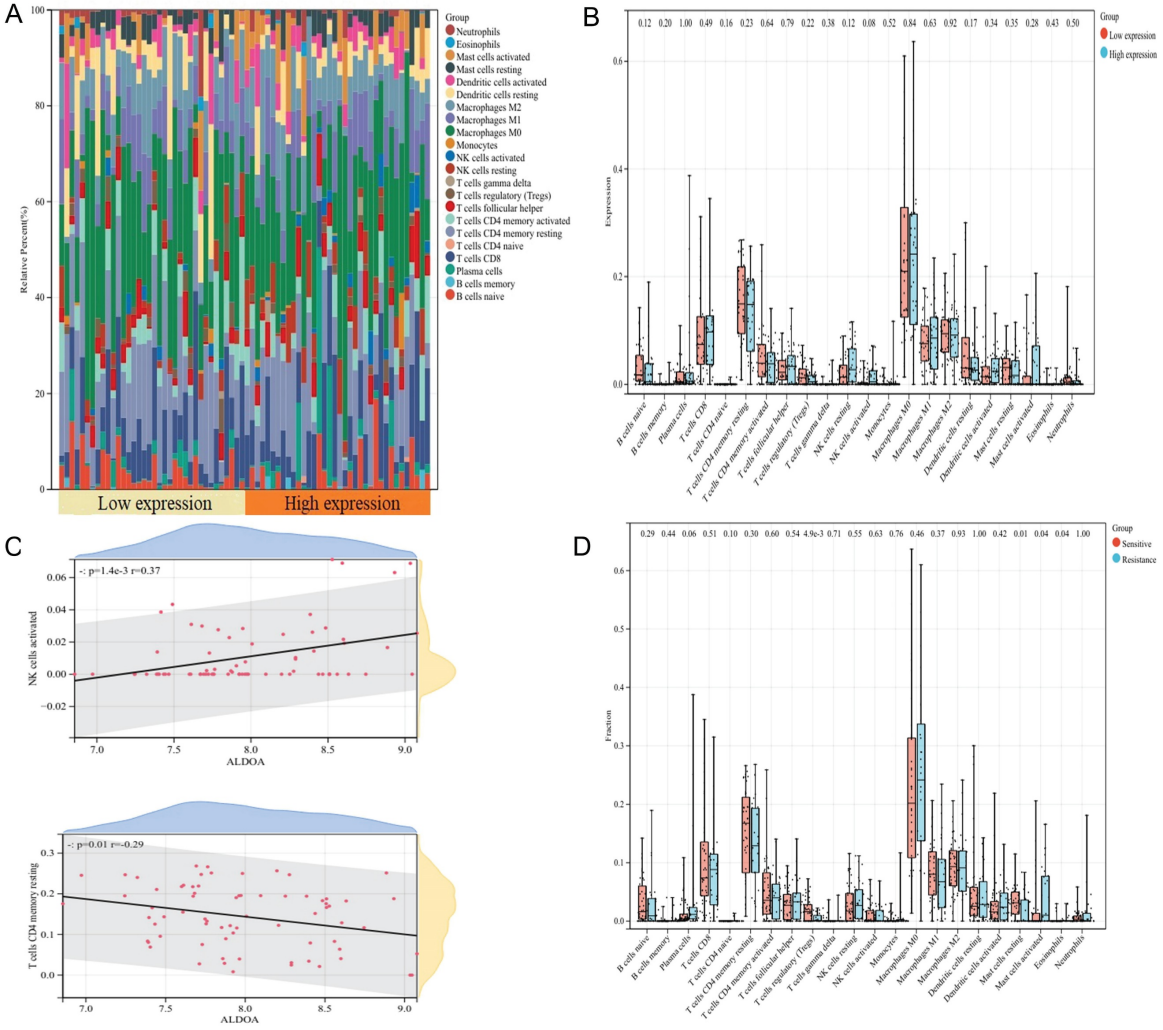
Stacked histogram of 22 kinds of immune cells in high and low expression group (A); Correlation between ALDOA expression and immune cell infiltration (B); Difference in the proportion of immune cell infiltration between high and low expression groups (C); Difference in the proportion of immune cell infiltration between sensitive group and drug resistant group (D).

We investigated the relationship between ALDOA expression and 22 kinds of immune cells by Cibersort method. According to the median expression of ALDOA in 72 patients, they were divided into low expression group and high expression group. We found that macrophages M0 accounted for the majority of immune cells in both groups (Fig. 7A), and there was no significant difference in the proportion of immune cell infiltration between the two groups (Fig. 7B). However, correlation analysis showed that the expression of ALDOA was positively correlated with the infiltration of NK cells activated immune cells (*P*=1.4e-3, r=0.37), and negatively correlated with T cells CD4 memory resetting (*P*=0.01, r=-0.29) (Fig. 7C). Meanwhile, we also analyzed the difference in the proportion of immune cell infiltration between the chemotherapy sensitive group and the chemotherapy resistant group, and found that there were differences in the four kinds of immune cell infiltration of T cells regulation (Tregs), mast cells resetting, mast cells activated and eosinophils between the two groups (Fig. 7D), indicating that these four kinds of immune cells may be related to the difference in chemotherapy response of oral cancer.

### Prognostic analysis and Immunohistochemical results

**Figure 8:**
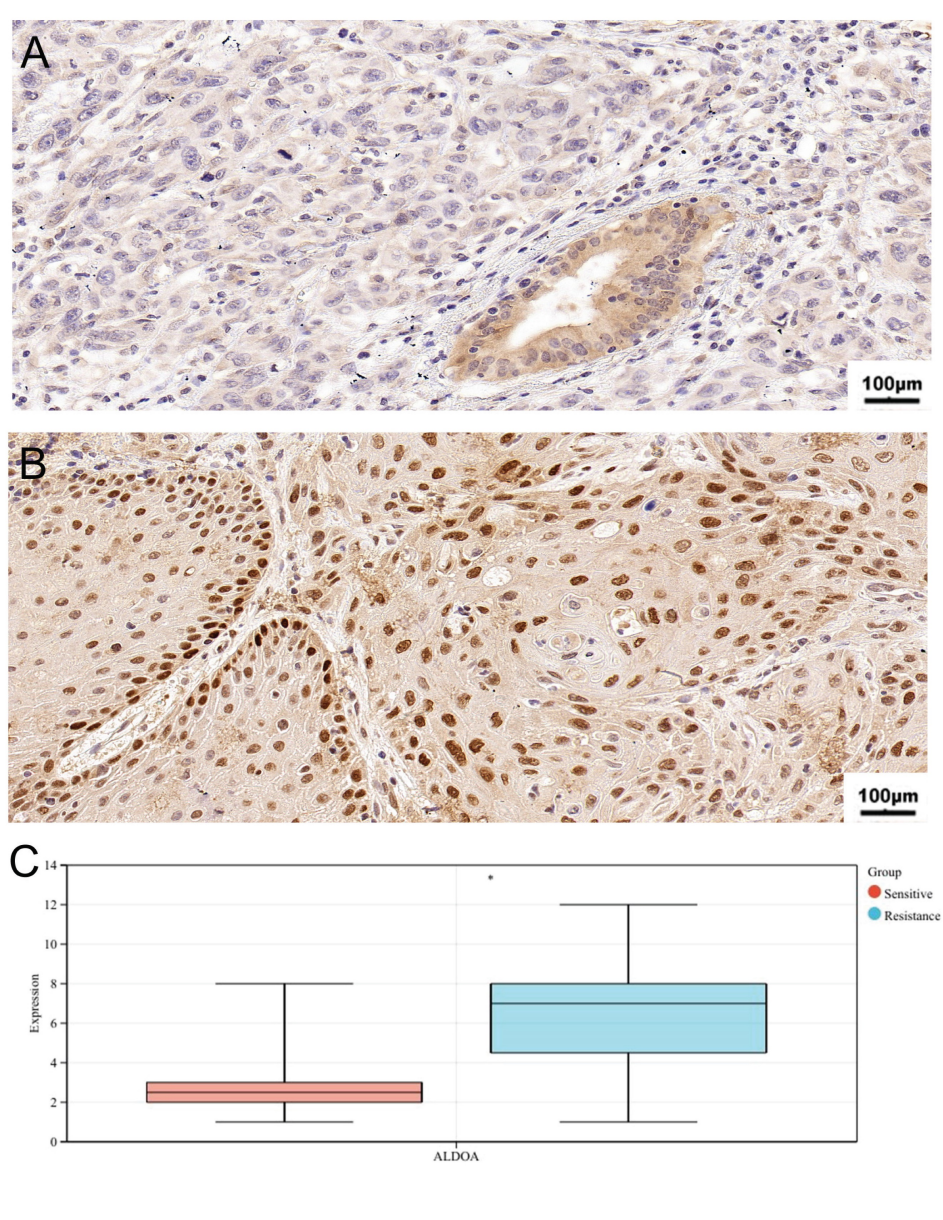
The representative images of ALDOA staining in Chemosensitivity (A) and chemoresistance tissues (B); IHC score of chemosensitivity and chemoresistance tissues (C). *P < 0.05, **P < 0.01, ***P < 0.001, ****P < 0.0001.

Table 2 shows the clinical characteristics of 21 patients with oral cancer. According to the immunohistochemical scoring standard, 8 patients' sections were positive and 13 patients' sections were negative. The average age of the negative group was 67.85 years old. Compared with the positive group, most patients in the negative group were in “T1+T2” stages and “N0+N1” stages, which were sensitive to chemotherapy and had a better prognosis. In terms of prognosis analysis, high-level ALDOA patients have high T stage, N stage and pathological stage, which indicates that high expression of ALDOA is associated with poor prognosis (Supplementary Figure 3). Similarly, through immunohistochemical analysis, we found that high expression of ALDOA is associated with chemotherapy resistance (Fig 8A,B,C). This indicates that the hypoxia gene ALDOA can be used as a predictor of the prognosis and chemotherapy efficacy of oral cancer.

## Discussion

Oral cancer is one of the common malignant tumors in the head and neck 21. Chemotherapy resistance is often an important factor leading to poor prognosis of patients with oral cancer 22. A large number of previous studies have confirmed that hypoxia is closely related to chemotherapy resistance 23. Based on bioinformatics methods, this study explored the hypoxia related genes that play an important role in chemotherapy resistance in patients with oral cancer.

Meanwhile, six chemotherapy response genes were screened by Univariate analysis. Based on gene expression profile and clinical data of chemotherapy response, a prediction model of chemotherapy response was constructed by using machine learning algorithm. The prediction performance of four machine learning algorithms was compared, and it was found that XGBoost algorithm had the best prediction performance. Meanwhile, this study also identified ALDOA as the core gene of this experiment. ALDOA belongs to aldolase family, which contains three different isozymes: ALDOA, ALDOB and ALDOC. In normal cells, glucose decomposes in the cytoplasm through glycolysis to produce a small amount of ATP, and further enters the mitochondria to complete the tricarboxylic acid cycle and oxidative phosphorylation, producing a large amount of ATP. However, most cancer cells tend to rely on glycolysis to rapidly generate ATP and biosynthetic precursors even when oxygen is sufficient. The up-regulated expression and enhanced activity of ALDOA promote this process, enabling tumors to quickly obtain energy and biosynthetic materials, supporting their rapid growth and invasive behavior 24. Recently, ALDOA was found to have nonenzymatic roles in drug resistance by interacting with different proteins 25-27. The relationship between ALDOA and chemotherapy resistance is mainly concentrated in colorectal cancer and breast cancer. At present, there is a scarcity of research on the relationship between ALDOA and chemotherapy resistance in oral cancer. This study confirmed that the high expression of ALDOA is associated with chemotherapy resistance in oral cancer by bioinformatics methods. Hypoxia gene ALDOA may be a potential target for the treatment of oral cancer. Targeting ALDOA can help to improve the chemotherapy response and prolong the survival time of patients.

However, there are still many deficiencies in this study. First, this study only confirmed the correlation between ALDOA overexpression and chemotherapy resistance by IHC. The mechanism of ALDOA induced chemotherapy resistance in patients with oral cancer needs to be further explored by *in vivo* and *in vitro* experiments. Second, this experiment needs to include more patients and follow-up to further analyze the survival and prognosis of patients, Finally, this experiment is a retrospective study, which needs to be further verified by a multi-center prospective study.

## Supplementary Material

Supplementary figures.

## Figures and Tables

**Table 1:** Clinical characteristics table.

Characteristics	Low expression (N=36)	High expression (N=36)	Total (N=72)	*P*-value
Age				
Mean±SD	56.42±12.61	58.50±8.61	57.46±10.77	
Sex				1.00
Female	7(9.72%)	8(11.11%)	15(20.83%)	
Male	29(40.28%)	28(38.89%)	57(79.17%)	
T stage				0.04
T1+T2	16(22.22%)	7(9.72%)	23(31.94%)	
T3+T4	20(27.78%)	29(40.28%)	49(68.06%)	
N stage				4.70e-03
N0+N1	25(34.72%)	12(16.67%)	37(51.39%)	
N2+N3	11(15.28%)	24(33.33%)	35(48.61%)	
M stage				1
M0	35(48.61%)	35(48.61%)	70(97.22%)	
MX	1(1.39%)	1(1.39%)	2(2.78%)	
Pathologic stage				0.37
Stage II+III	9(12.50%)	5(6.94%)	14(19.44%)	
Stage IV	27(37.50%)	31(43.06%)	58(80.56%)	
Chemotherapy response				0.09
Sensitive	25(34.72%)	17(23.61%)	42(58.33%)	
Resistance	11(15.28%)	19(26.39%)	30(41.67%)	

**Table 2:** Clinical characteristics of 21 patients with oral cancer.

Characteristics	Positive(N=8)	Negative(N=13)	Total(N=21)	*P*-value
Age				
Mean ± SD	61.50±9.26	67.85±8.58	65.43±9.17	
Sex				1.00
Female	2(9.52%)	4(19.05%)	6(28.57%)	
Male	6(28.57%)	9(42.86%)	15(71.43%)	
T stage				0.02
T1+T2	1(4.76%)	10(47.62%)	11(52.38%)	
T3+T4	7(33.33%)	3(14.29%)	10(47.62%)	
N stage				0.02
N0+N1	4(19.05%)	13(61.90%)	17(80.95%)	
N2+N3	4(19.05%)	0(0.0e+0%)	4(19.05%)	
M stage				
M0	8(38.10%)	13(61.90%)	21(100.00%)	
Pathologic stage				0.08
Stage II+III	1(4.76%)	8(38.10%)	9(42.86%)	
Stage IV	7(33.33%)	5(23.81%)	12(57.14%)	
Chemotherapy response				0.03
Resistance	5(23.81%)	1(4.76%)	6(28.57%)	
Sensitive	3(14.29%)	12(57.14%)	15(71.43%)	
