# Supplementary material for: Establishment of Chemotherapy Prediction Model Based on Hypoxia-Related Genes for Oral Cancer
Source: J Cancer. 2024 Aug 13;15(16):5191–203. doi: 10.7150/jca.96654 (PMC11375540; doi:10.7150/jca.96654)
Supplement: Supplementary file 1 — Supplementary figures. [file jcav15p5191s1.pdf]

Supplementary materials

Figure 1: The ROC curves of decision trees, support vector machines, random forests, and XGBoost validation groups are shown in the top left, top right, bottom left, and bottom right, respectively.

Figure 2: Expression of ALDOA in head and neck cancer cell lines(A); Expression of VEGFA in head and neck cancer cell lines(B).

Figure 3: The relationship between ALDOA expression and prognosis: T stage , N stage, pathological stage.

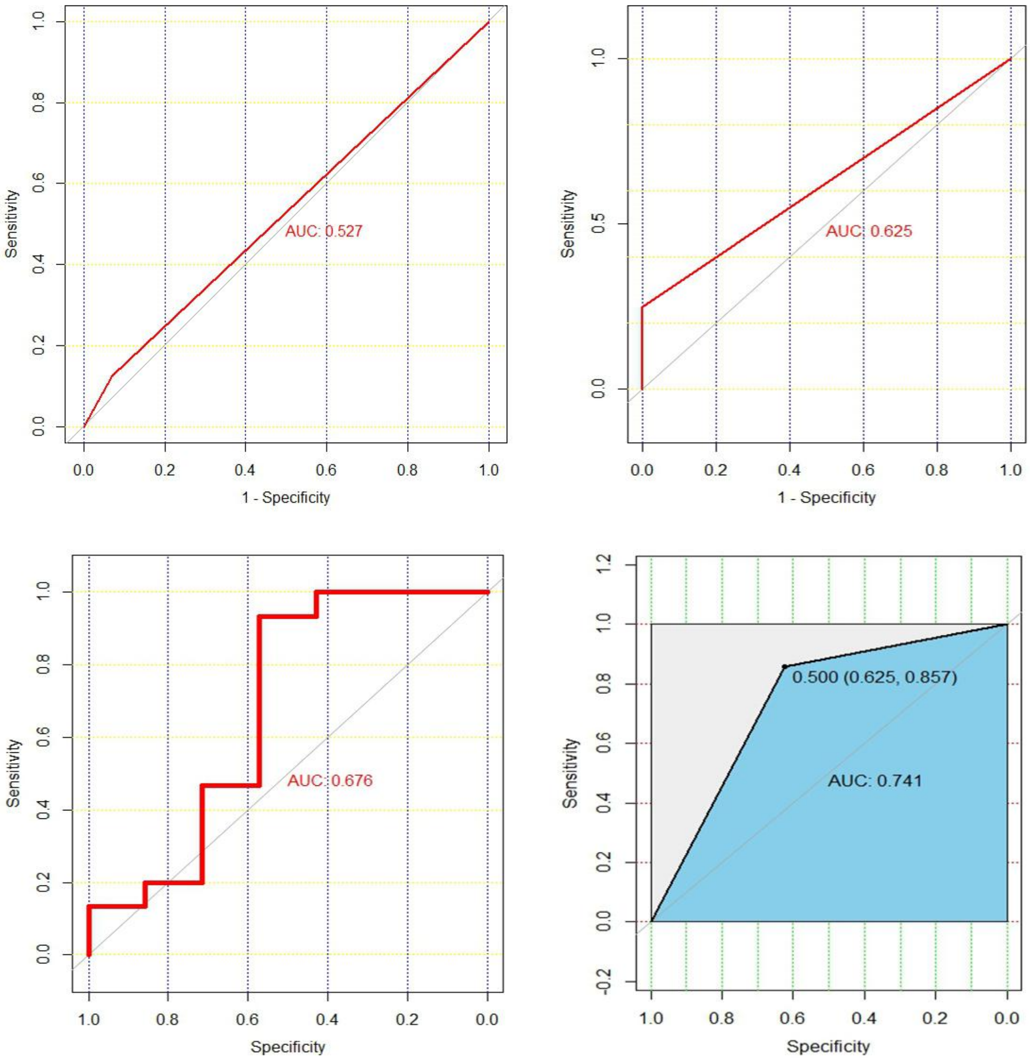

Figure 1:The ROC curves of decision trees, support vector machines, random forests, and XGBoost validation groups are shown in the top left, top right, bottom left, and bottom right, respectively.

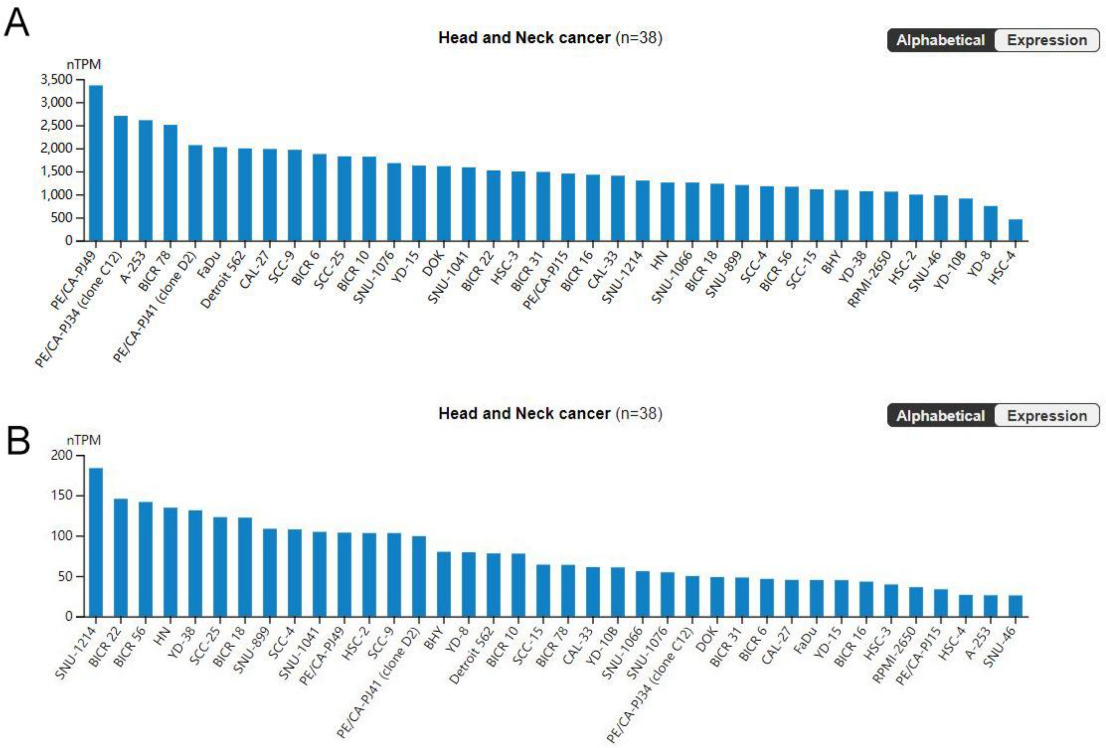

Figure 2: Expression of VEGFA in head and neck cancer cell lines.

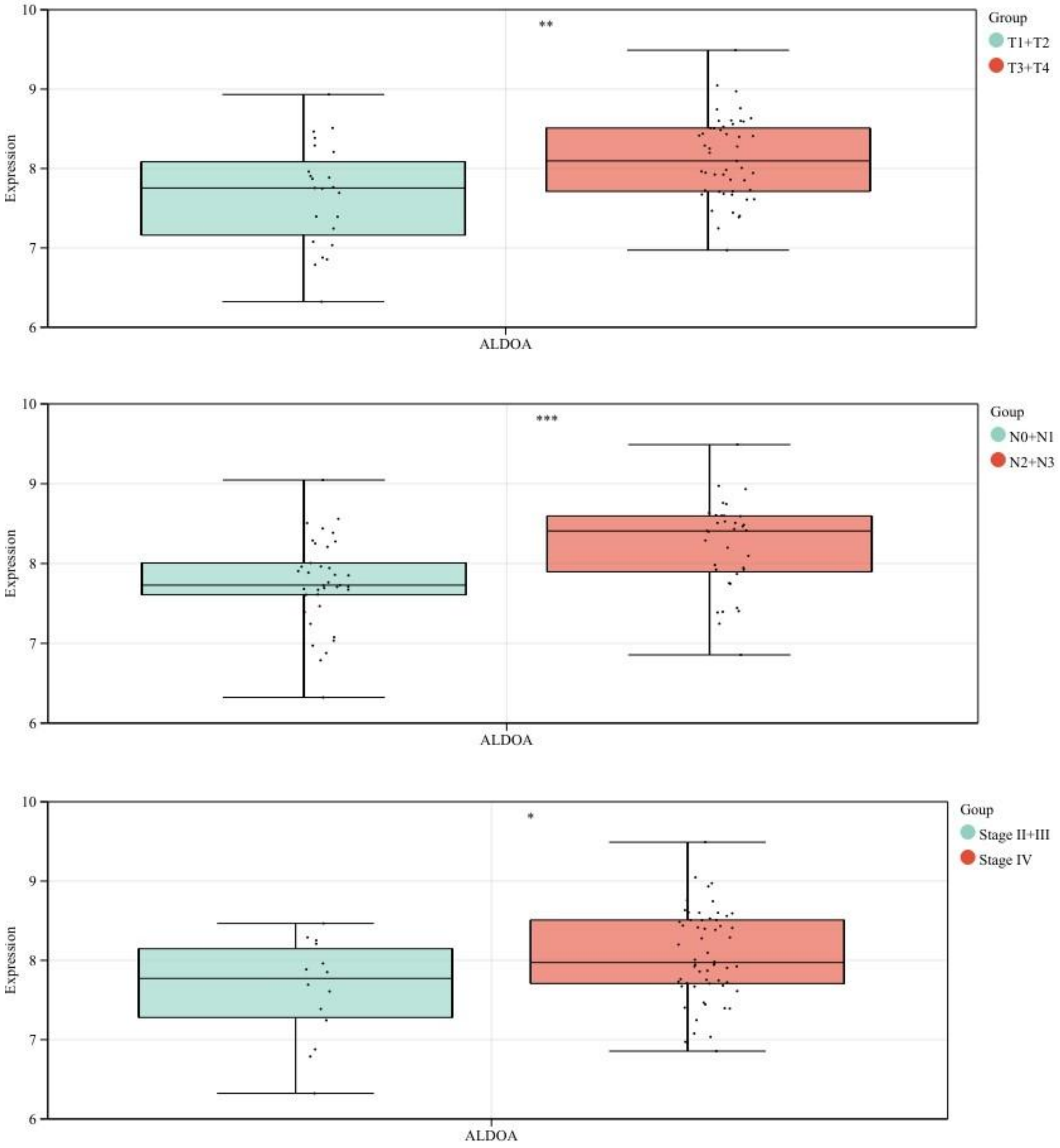

Figure 3:The relationship between ALDOA expression and prognosis: T stage ,N stage, pathological stage. *P < 0.05， **P < 0.01， ***P < 0.001， ****P < 0.0001.
